# Supplementary material for: Position- and scale-invariant object-centered spatial localization in monkey frontoparietal cortex dynamically adapts to cognitive demand
Source: Nat Commun. 2024 Apr 18;15:3357. doi: 10.1038/s41467-024-47554-4 (PMC11026390; doi:10.1038/s41467-024-47554-4)
Supplement: Supplementary file 1 — Supplementary Information [file 41467_2024_47554_MOESM1_ESM.pdf]

Supplementary information

Position- and scale-invariant object-centered spatial localization in monkey frontoparietal cortex dynamically adapts to cognitive demand

Bahareh Taghizadeh[1,2], Ole Fortmann[1,3], *Alexander Gail[1,3,4,5]

[1] Sensorimotor Group, German Primate Center, Göttingen, Germany

[2] School of Cognitive Science, Institute for Research in Fundamental Sciences (IPM), P.O. Box 19395-5746, Tehran, Iran

[3] Faculty of Biology and Psychology, University of Göttingen, Göttingen, Germany

[4] Bernstein Center for Computational Neuroscience, Göttingen, Germany

[5] Leibniz ScienceCampus Primate Cognition, Göttingen, Germany

Corresponding author: Alexander Gail (agail@gwdg.de)

Table of contents

Supplementary Figure 1	3
Supplementary Figure 2	4
Supplementary Figure 3	5
Supplementary Figure 4	8
Supplementary Figure 5	9
Supplementary Figure 6	10
Supplementary Figure 7	11
Supplementary Figure 8	13
Supplementary Note 1	14
Supplementary Figure 9	16
Supplementary Figure 10	18
Supplementary Note 2	18
Supplementary Table 1	20
Supplementary References	21

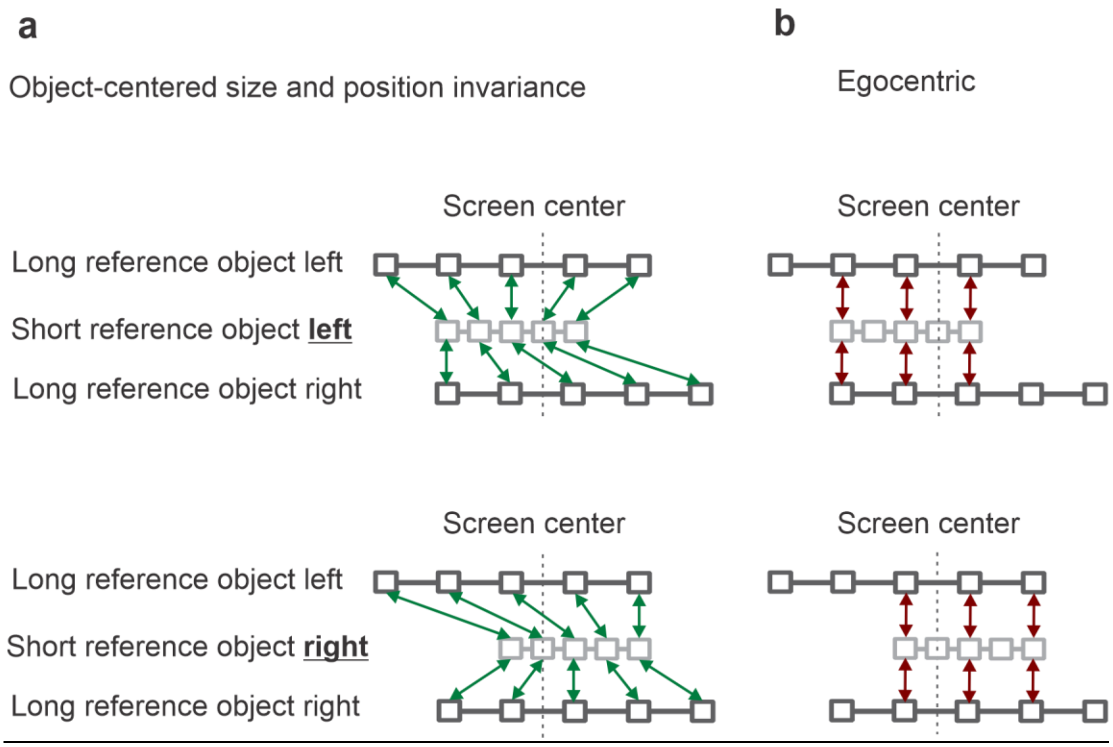

Supplementary Figure 1. Corresponding position information for calculating Position and Size Invariance (PSI) between different task conditions. a Corresponding positions for calculating alloCorr. Invariance with respect to object position and object size predicts similar neural selectivity profiles between short-object and long-object conditions when the profile is computed relative to the within-object numerical box positions (1, 2, 3, …). To calculate alloCorr, we considered all possible correspondences (green arrows) between long and short objects when the short objects presented on the left (top panels) or right (bottom row) of the screen center (vertical dotted line). Box 1 (2, 3, …) of the object here always corresponds to box 1 (2, 3, …), irrespective of object position and size. We correlated a vector of 20 firing rates from long-object selectivity profiles with a vector of 20 firing rates from short-object profiles. Higher alloCorr results in higher PSI. **b** Corresponding positions for calculating egoCorr. The egocentric hypothesis predicts similar activity profiles for boxes between long and short objects which share the same position on the screen (= relative to the body). Accordingly, to calculate egoCorr, we considered every possible overlap (red arrows) between long and short objects when objects presented on the left (top row) or right (bottom row) of the screen center (vertical dotted line). We correlated a vector of 10 firing rates from long-object selectivity profiles with a vector of 10 firing rates from short-object profiles. Higher egoCorr results in lower PSI.

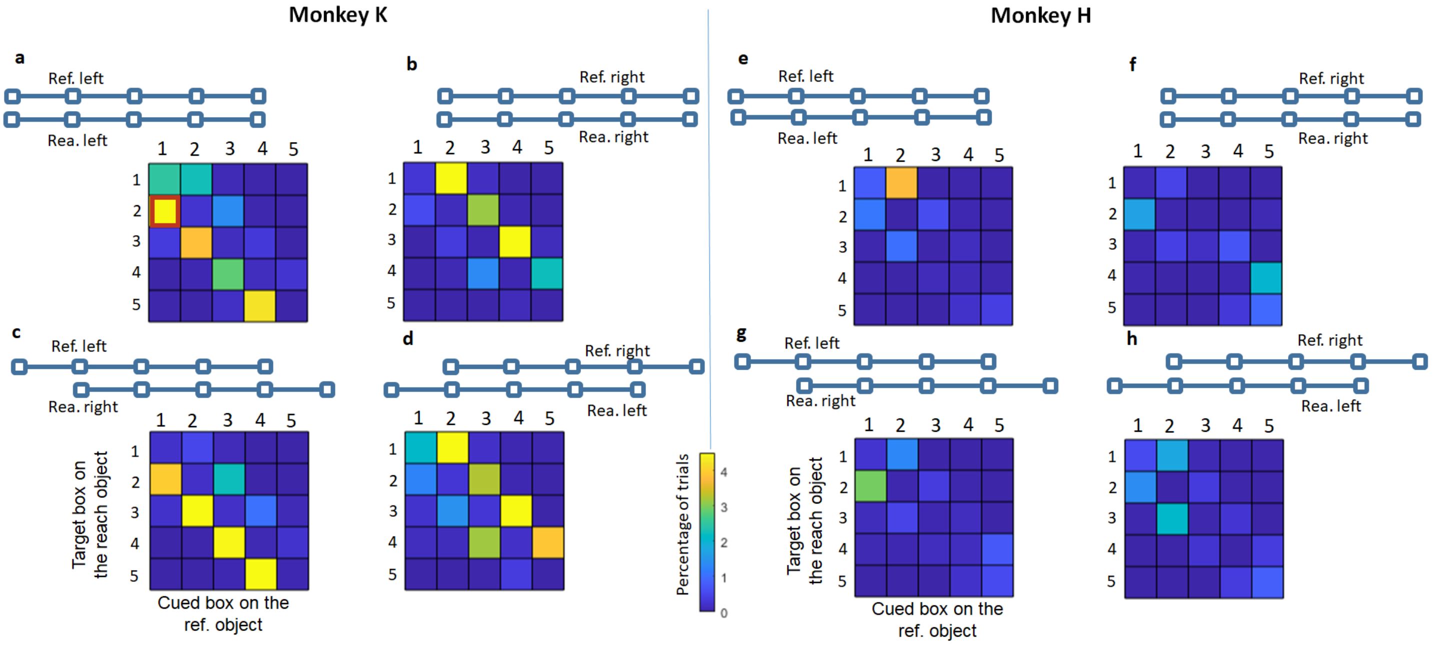

Supplementary Figure 2. **Error matrices of behavioral performance in Exp I.** The error matrices show the average across-session percentage of incorrect trials for different cued boxes on the reference object, plotted for different combinations of reference and reach object positions (reference and reach object position in **a** and **e**: left, left; **b** and **f** right, right; **c** and **g**: left, right; **d** and **h**: right, left), separately for the two monkeys (monkey K: panels **a**-**d**; monkey H: panels **e**-**h**). In every error matrix, columns represent cued box on the reference object and rows represent target box on the reach object. Percentage of trials was calculated relative to the number of trials with that particular task condition, not relative to all trials. For example, in panel a, the element of the matrix which is marked by a red square indicates percentage of trials when box #1 (counting from left to right) on the reference object was cued but the monkey touched the second box on the reach object, across all trials where both reference and reach objects were on the left side and box number #1 was cued. The main diagonal of the error matrices indicate percentage of trials where the monkey touched the correct box on the reach object but initiated the movement later than the permitted time (time-out error). The pattern of errors show that monkey K's reach movement in his error trials was biased towards the center of the screen, probably because it was closer to the eye fixation point and was easier for him to reach to the target with high spatial precision. Monkey H in general made errors for left most targets on the object, confusing boxes #1 and #2 especially when the reference object was on the left side. Given the small size of the cue, this probably happened because he could not precisely see on which box the cue was located in its left most positions.

a

Monkey K, PMd

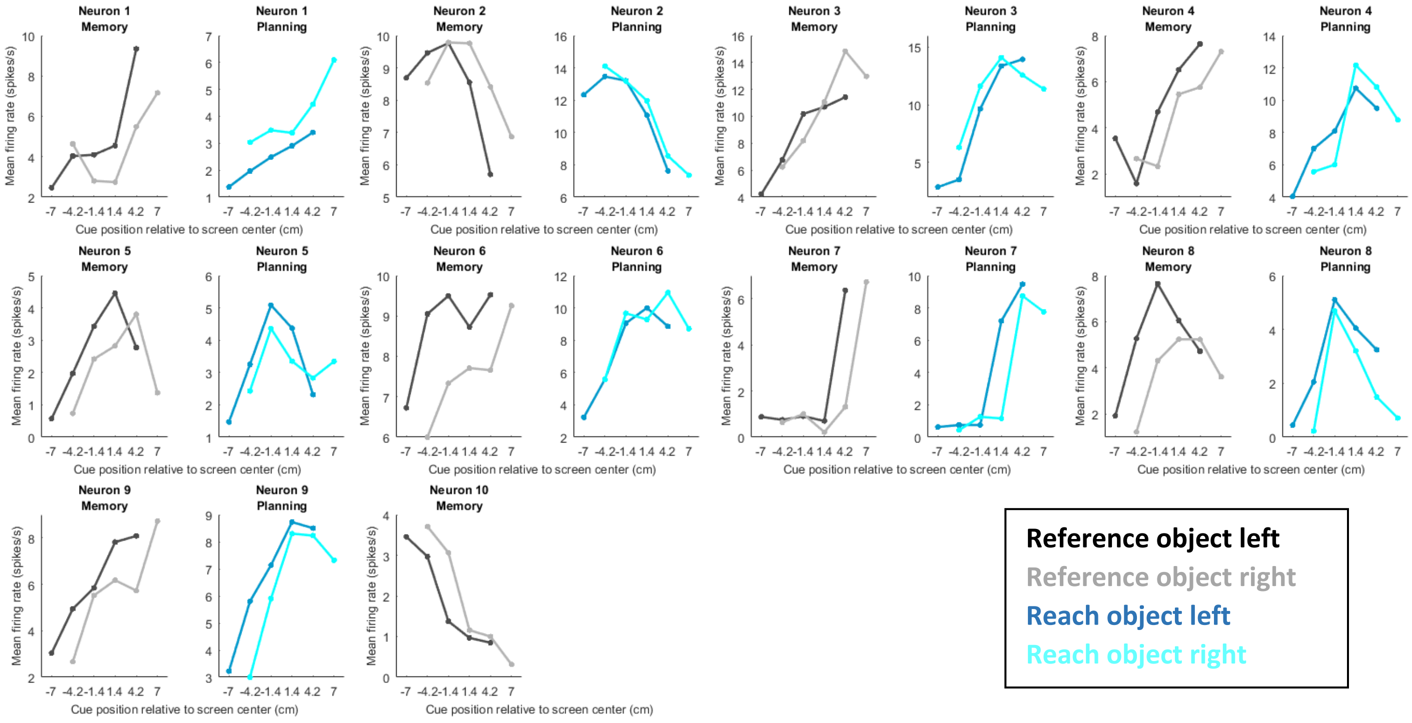

b

Monkey K, PRR

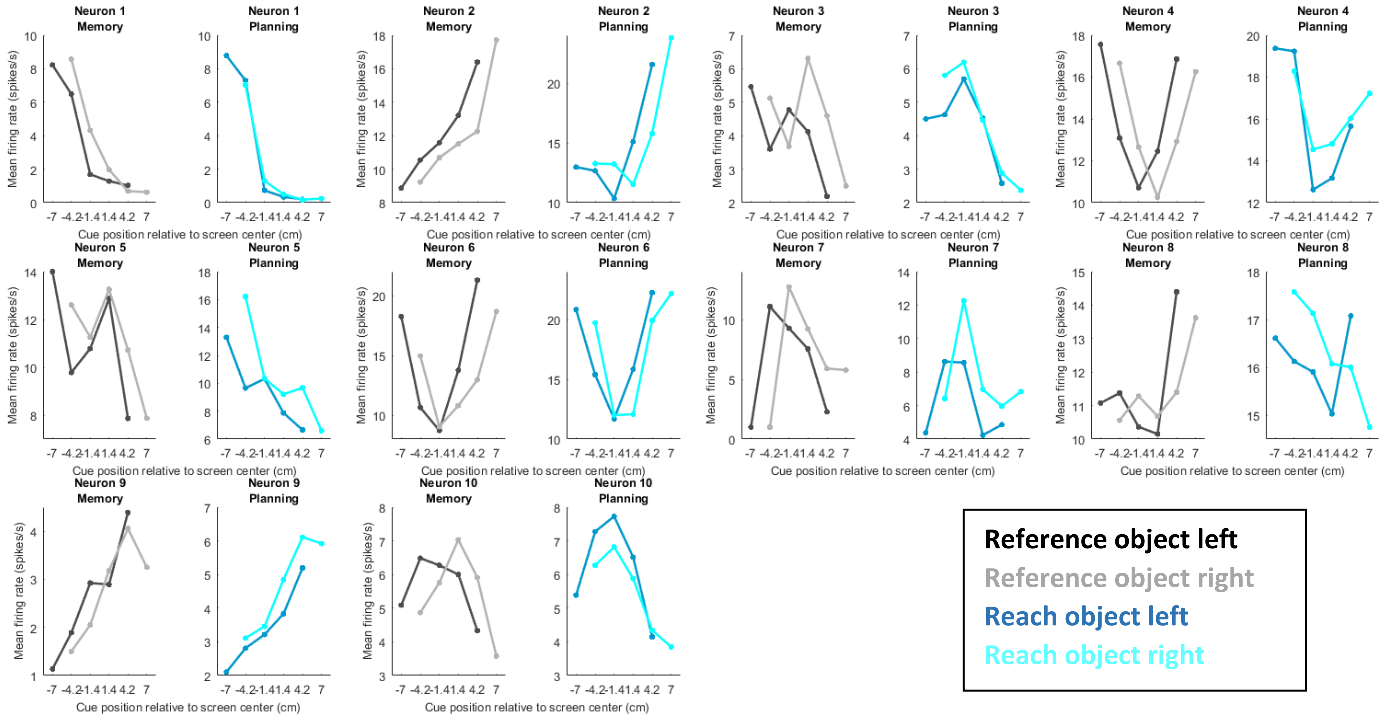

c

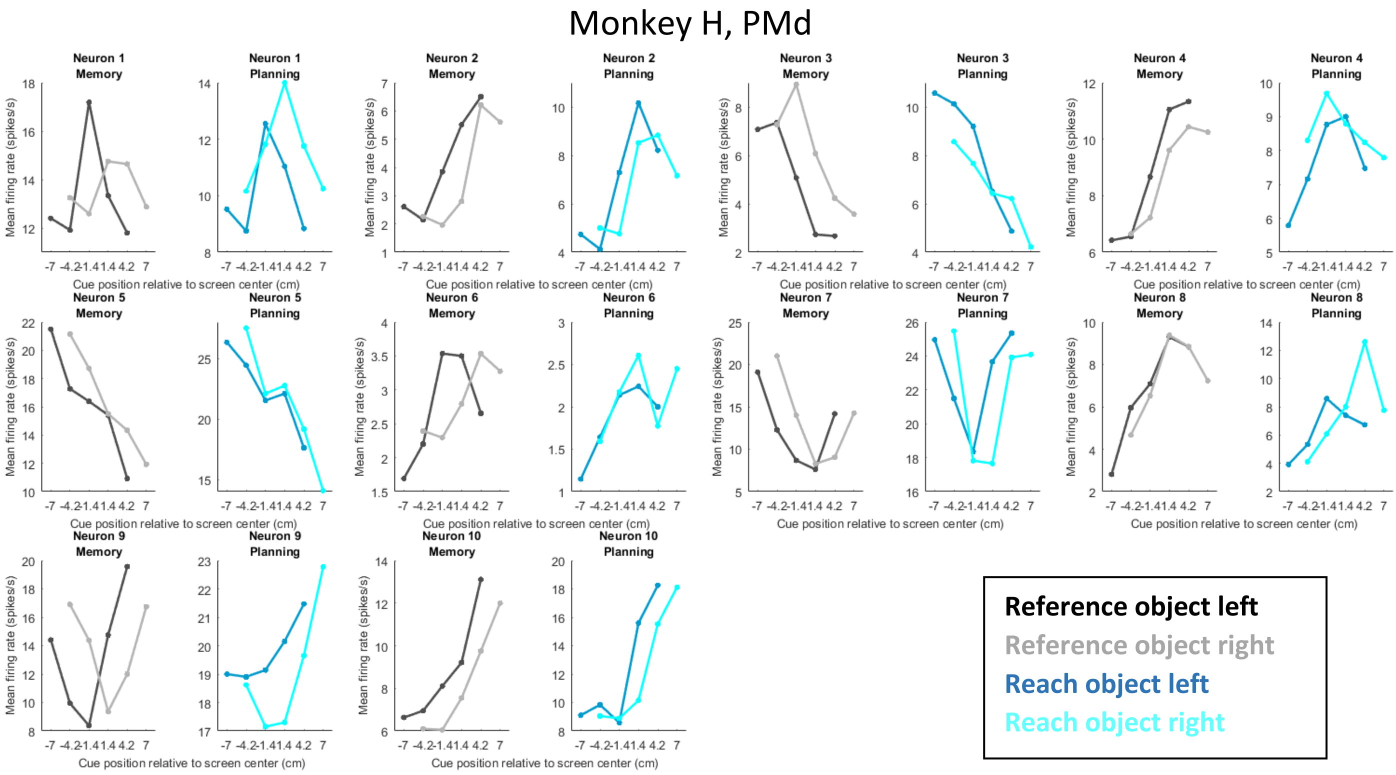
Monkey H, PMd
Mean firing rate (spikes/s)
Cue position relative to screen center (cm)
Reference object left
Reference object right
Reach object left
Reach object right

d

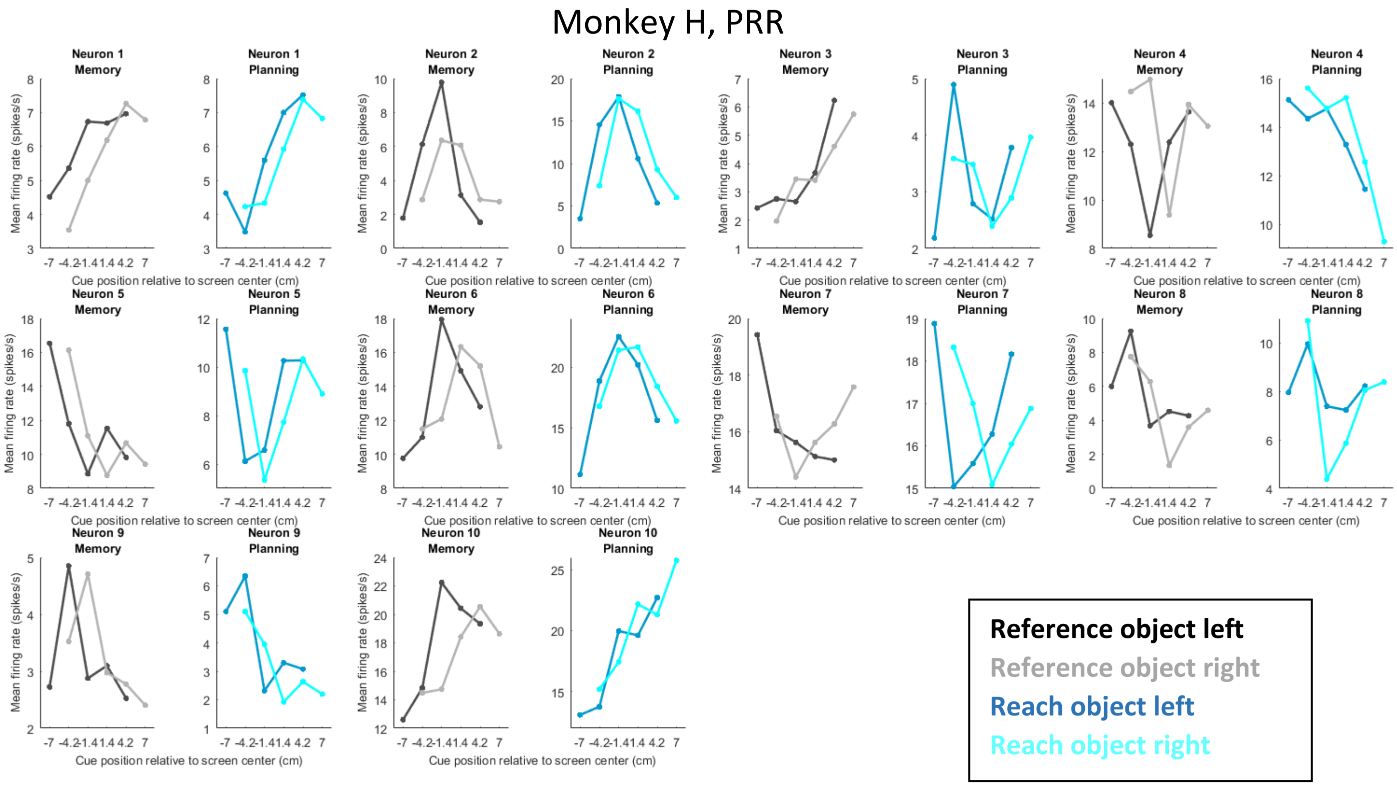
Monkey H, PRR
Mean firing rate (spikes/s)
Cue position relative to screen center (cm)
Reference object left
Reference object right
Reach object left
Reach object right

Supplementary Figure 3. Selectivity profile of more example neurons. Selectivity profile of 10 example neurons each in **a** monkey K, PMd, **b** monkey K PRR, **c** monkey H PMd, **d** monkey H PRR. For every neuron, light and dark gray curves show selectivity profile to 5 cue positions on the reference object, in last 300ms of the memory period, when the reference object was on the left and right, respectively; light and dark blue curves show selectivity profile to 5 target positions on the reach object, in last 300ms of the movement planning period, when reach object was on the left and right, respectively. The value of the objCorr, egoCorr and PI for every neuron is given in the tables.

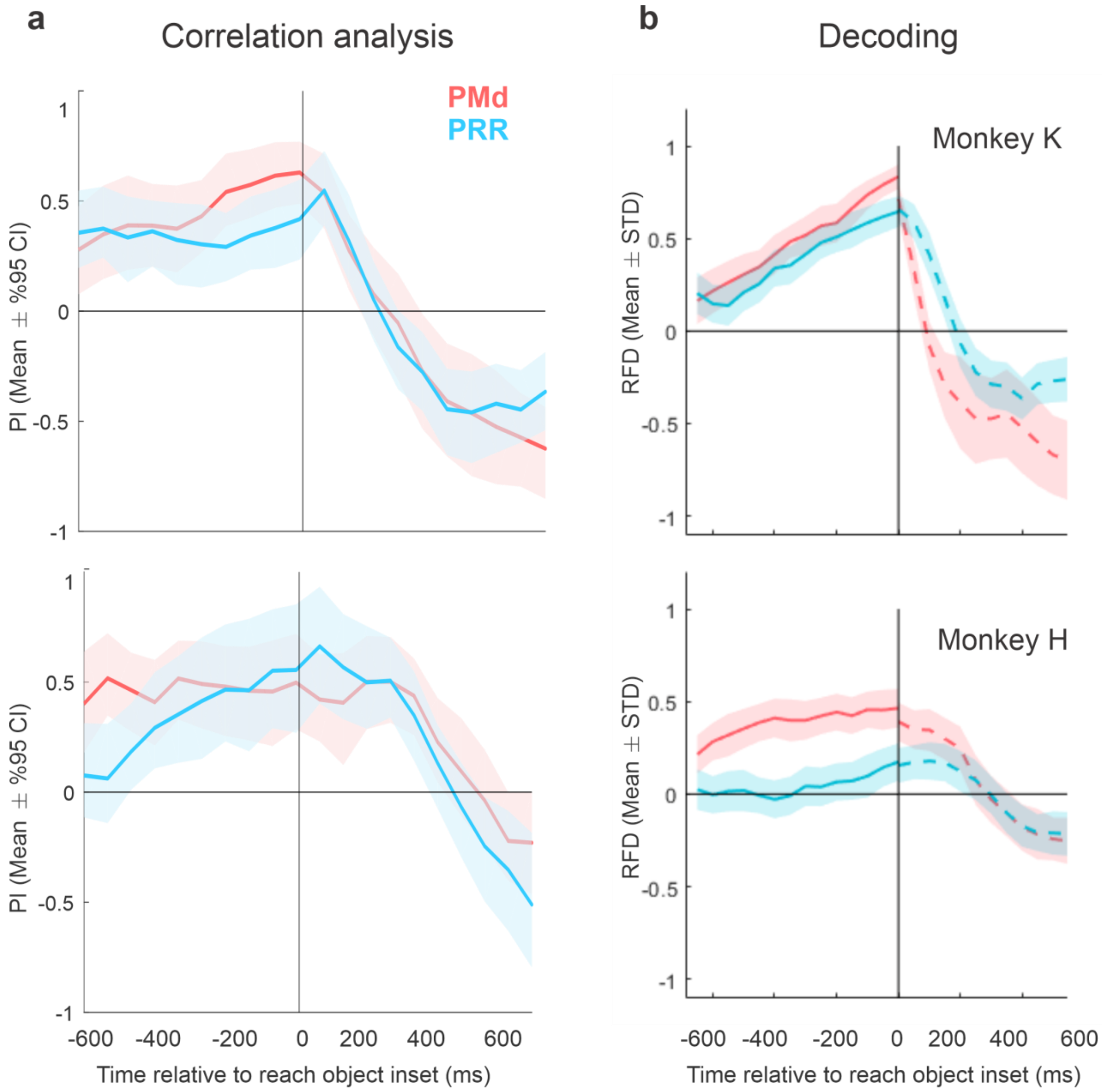

Supplementary Figure 4. Preferred reference frame across neuronal population. a PI measure from the correlation analysis (mean and 95% confidence interval across N=100 bootstrap samples for all cases of monkey and area) and **b** the RFD from the decoding analysis for monkeys K and H. All settings are as in Figure 3. Both monkeys show dominant object-centered encoding in late memory period and egocentric encoding in late planning period. The transition of the reference frame is faster in monkey K compared to H.

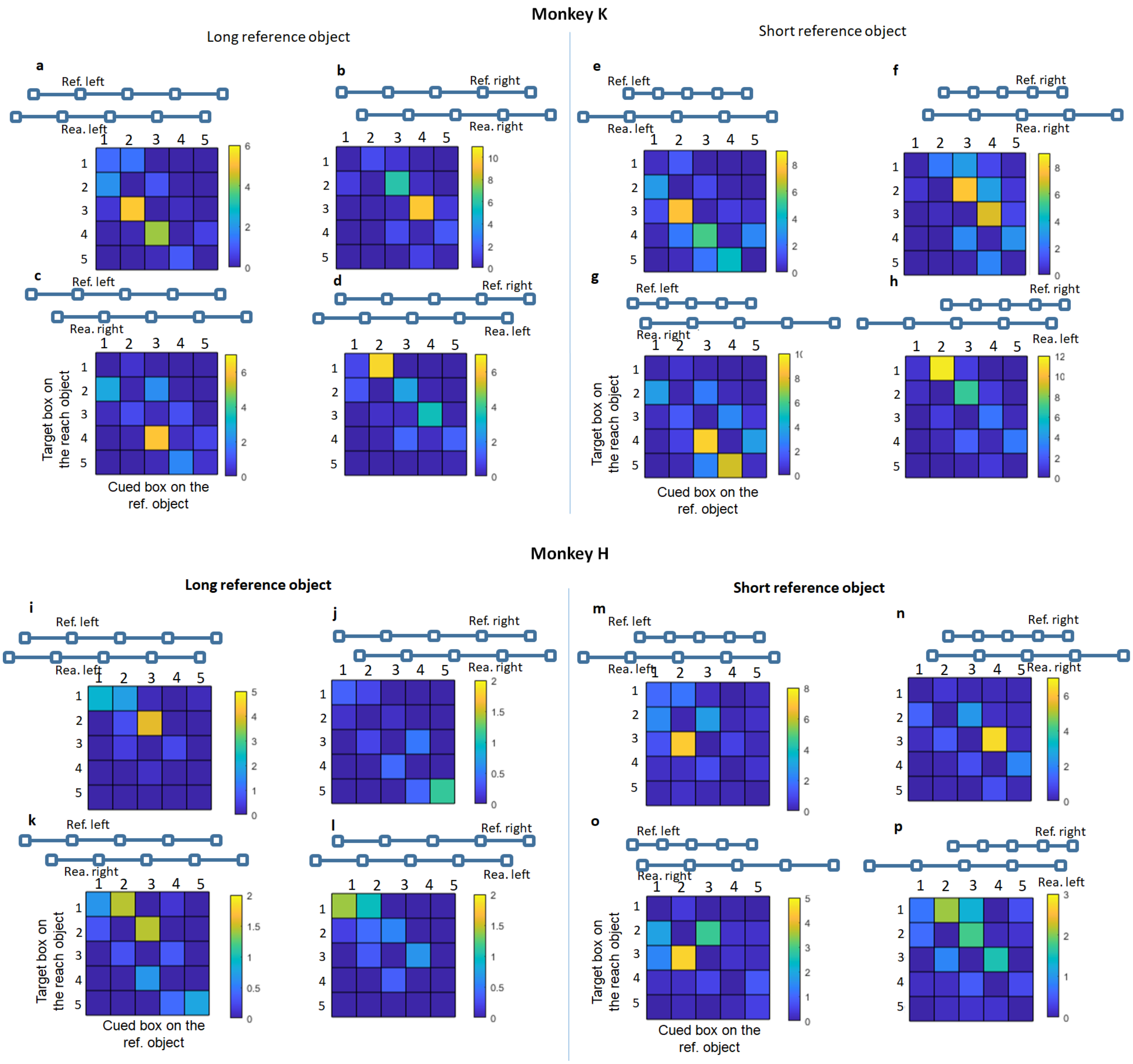

Supplementary Figure 5. Error matrices of behavioral performance in Exp II. The error matrices show average-across-session percentage of incorrect trials for different cued boxes on the reference object, plotted for different combinations of reference and reach object sizes and positions (long reference object and reach object position in **a, e, i** and **m**: left, left; **b, f, j** and **n** right, right; **c, g, k** and **o**: left, right; **d, h, l** and **p**: right, left), separately for the two monkeys (monkey K: panels **a**-**h**; monkey H: panels **i**-**p**). The rest of the settings are as explained in Supplementary Fig. 2. In general both monkeys showed same pattern of error as in Exp I. Here monkey H shows more bias towards the center as compared to Exp I, especially when the short reference object was on the right, **n** and **p**.

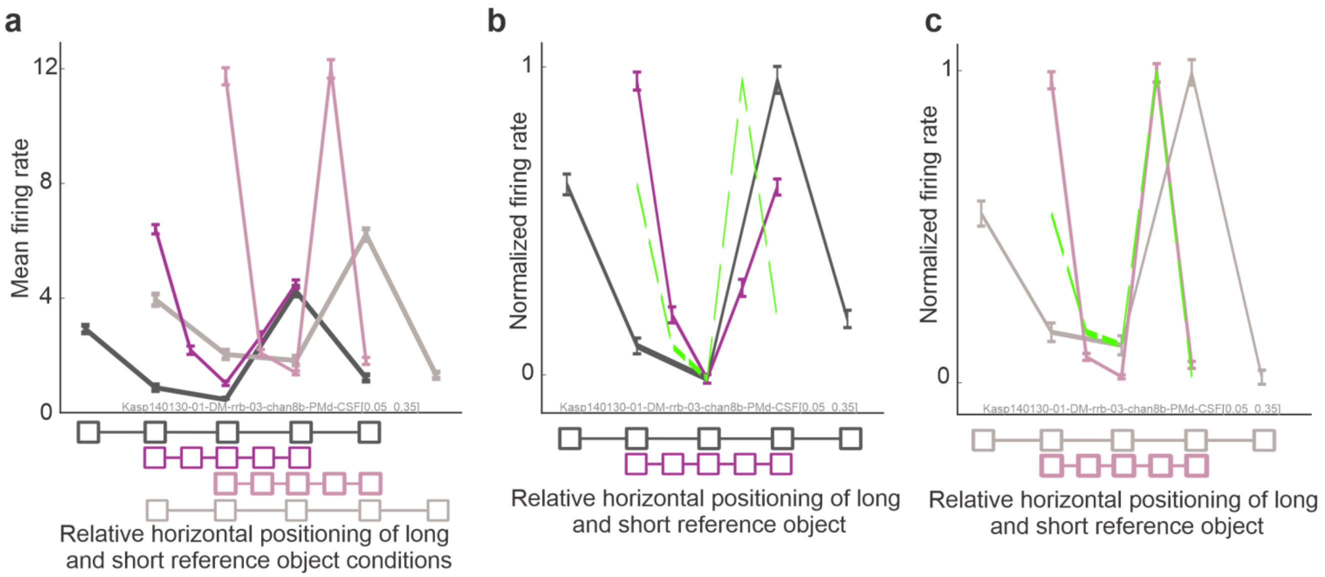

Supplementary Figure 6. Example unit with mixed reference frame in Exp II. a Selectivity profiles of a PMd unit in early memory period, 50 – 350 ms after cue offset, for different object size and positions. **b** and **c** show normalized version of the same selectivity profiles as in **a**, separately for long and short objects left **b** and right **c**. The green dashed curves show the predicted selectivity profile for the short object trials based on the selectivity profiles in the long object trials, assuming ideal object-centered encoding (i.e., the squeezed version of the long object selectivity profiles relative to the central box of the object). For this unit, selectivity profiles in long-object-left (dark grey) and long-object-right (light grey) indicate position invariance. Also, the short-object-right (light purple) selectivity profile is quite close to a horizontally scaled version of the long-object profile, with gain modulation. In this sense, this unit meets predictions of a position- and size-invariant object-centered reference frame. However, the short-object-left profile deviates from the prediction, suggesting some form of mixed reference frame.

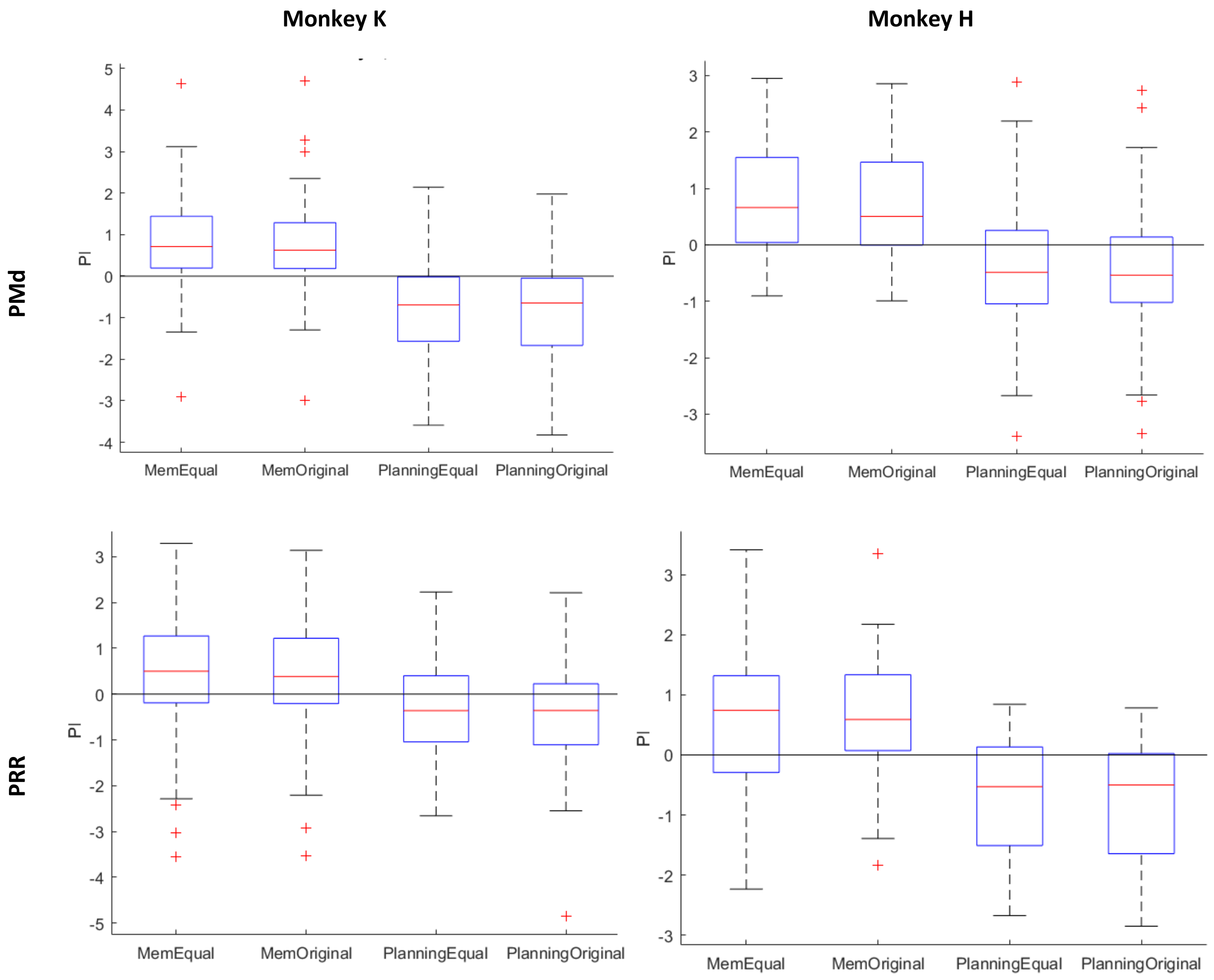

Supplementary Figure 7. Control analysis for number of task conditions included in calculating correlation coefficients in Exp I. The fact that the number of task conditions is higher for calculating the object-centered compared to the egocentric correlation coefficients could potentially create a confound. Since correlation coefficients may generally be biased towards smaller values when computed across fewer conditions, this could potentially induce a positive bias on PI and PSI values. To rule out that our results suffer from such bias, we ran a control analysis in which we balanced the number of task conditions across the object-centered and egocentric correlation coefficients. In Exp I, where 5 pairs of task conditions were used for calculating objCorr and 4 pairs for egoCorr, we calculated objCorr by randomly selecting 4 out of 5 pairs, and averaged across all possible selections. In the figure, this is indicated by MemEqual for the last 300 ms of the memory period, and by PlanningEqual for the last 300 ms of planning period. We then compared the PI value with unequal number of pairs (5 and 4 respectively for objCorr and egoCorr), in the figures indicated by MemOriginal and PlanningOriginal. There was no significant difference between the two ways of calculating PI (ranksum test

comparing MemEqual with MemOriginal, and, PlanningEqual with PlanningOriginal, separately for every monkey and brain area; all $p>0.5$). The box plots show median and the 75th (top) and 25th (bottom) percentiles, as well as the data range (whiskers) without putative outliers (red crosses; more distant from 25/75 percentiles than 1.5 times the respective interquartile range). Number of samples for monkey K N_{PMd}=77, N_{PRR}=71 ; monkey H N_{PMd}=32, N_{PRR}=30.

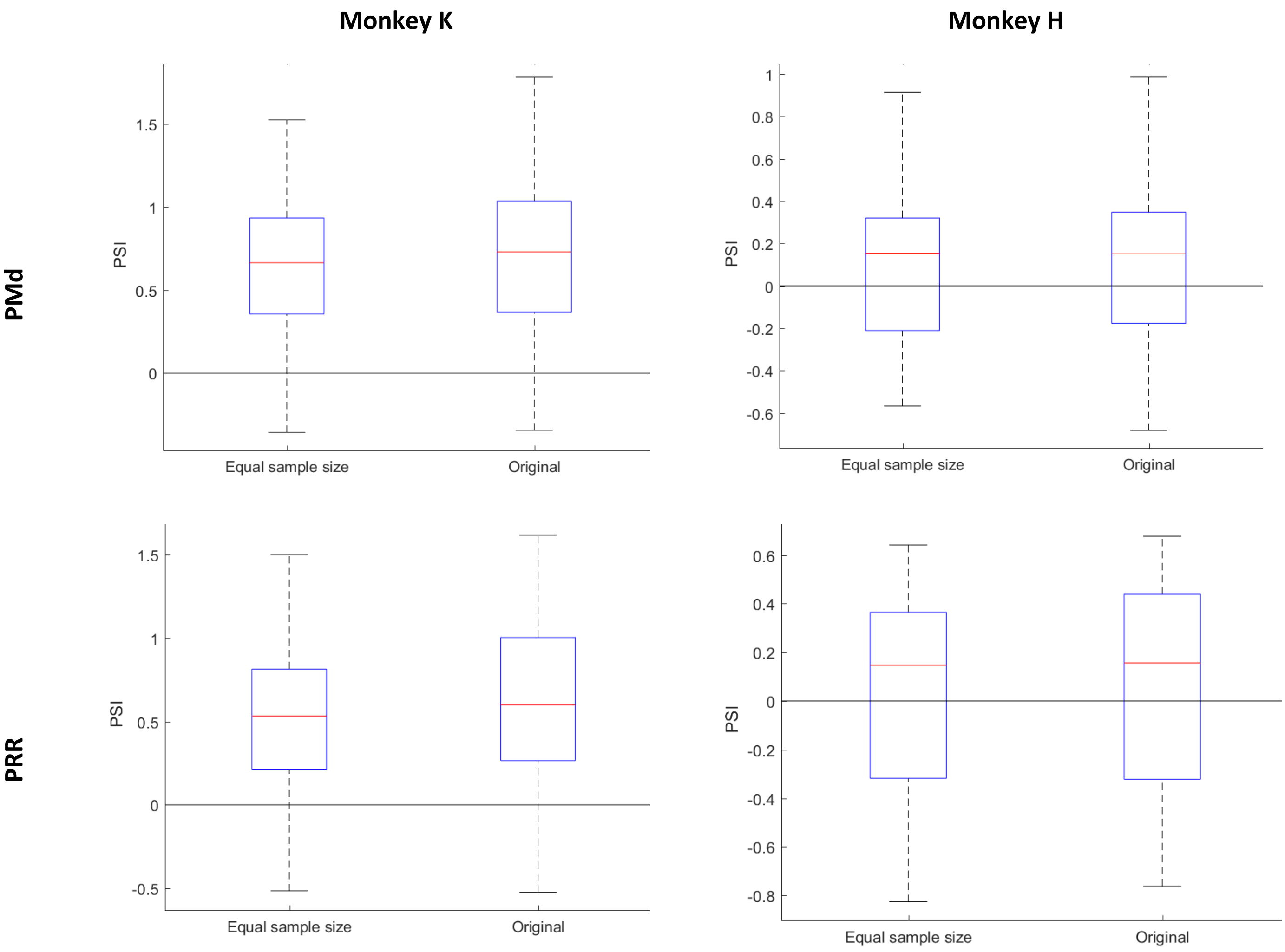

Supplementary Figure 8. Control analysis for number of task conditions included in calculating correlation coefficients in Exp II. Box plots show the PSI when number of task conditions was balanced for calculating alloCorr and egoCorr with the same strategy as in Supplementary Fig. 7 for the last 500 ms of the memory period. There was no significant difference between the two ways of calculating PSI (ranksum test, all $p>0.3$). The distribution of PSI values for both areas in monkey K were significantly shifted towards positive values (signed rank test, PRR and PMd $p < 0.001$. In monkey H, the same trend towards positive values did not reach significance when analyzing the same time bin (signed rank test, PMd $p=0.23$ and PRR $p=0.42$). Instead, monkey H showed stronger effect earlier during the memory period. During the time window [-850 -500] ms before the reach object onset, the distribution of PSI had a significantly positive shift in both areas (signed rank test on time bins of 300ms sliding by 50ms, PRR and PMd $p < 0.034$, data not shown).

Supplementary Note 1: Demixed principle component analysis of Exp II

Complementary to the PSI and RFD measures, we assessed position and size invariant encoding for the individual monkeys in Exp II with a demixed principal component analysis (dPCA)[1]. The dPCA is a dimensionality reduction method in which different components capture co-variation of the neural activity with different task parameters. The resulting components act as decoders that demix the dependency of the population activity to task parameters. For the analysis, we used the dPCA package provided by Kobak et al. 2016 (https://github.com/machenslab/dPCA).

The average firing rate of neurons from each monkey and area in different time bins of the memory period was formatted as a matrix of size [number of neurons x 2 x 5 x 2 number of time bins], where 2, 5 and 2 represents 2 object locations, 5 cue locations on the object and 2 object sizes, respectively; 9 time bins were included in this analysis starting from [-700 -400] ms to [-300 0] ms relative to reach-object onset, sliding by 50 ms. Accordingly, we quantified different categories of principal components (PCs): task-independent components (reflecting the time dimension), components that depend on the three task parameters of object size, object location and object-centered cue location (cue-dependent components), and components that depend on the interaction of task parameters.

Every PC resulting from the dPCA depends on one task parameter or their interactions. The percentage of explained variance in the subspace generated by cue-dependent PCs was for monkey K, PRR 88.6%, PMd 88.9%; monkey H, PRR 59.2%, PMd 44.5%. We restricted the rest of the analysis to the first 10 PCs, which in all four cases of monkey and brain areas captured at least 78% of the total variance of the data.

The results of the dPCA analysis show size invariant (object-centered) neural encoding in both animals and brain areas. When projecting the population activity onto the PC space, the first 3 cue-dependent PCs clustered the data into 5 clusters. The clusters were determined almost exclusively by the location of the cue on the object and were independent of object size and location (Supplementary Fig. 9a). We compared the quality of clustering for this object-centered approach with the quality when clustering task conditions based on the 9 possible egocentric locations of the cue. The 9 clusters then represent the 9 positions relative to the monkey, positions where the boxes on the object, and hence the cue, can be located along the horizontal axis. The number of combinations of object size and object position for the 9 different clusters varies then between 1 and 4. Since egocentric clustering may not happen only in the cue-dependent subspace but could also occur in other task parameter subspaces (which may or may not overlap with the cue subspace), we defined a clustering index (CI), to quantify how well each subspace separates the different task conditions.

The clustering index was defined as

(1) $$CI = \frac{\text{within cluster variance (W)}}{\text{between clusters variance (B)}}$$

Before calculating the variance, the neural trajectories were averaged across time bins for every task condition.

For every cluster C_k, the within-cluster variance (W_k) was calculated as

(2) $$W_k = \sum_{x_i \in C_k} ||x_i - \bar{x}||^2$$

Where x_i are the samples in the cluster and $\bar{x}$ is the cluster centroid. The total within-clusters variance was the sum of all within-cluster variances:

(3) $$W = \sum_{k=1}^{\text{number of clusters}} W_k$$

The between-clusters variance (B) was calculated as

(4) $$B = \sum_{k=1}^{\text{number of clusters}} ||\overline{X_k} - \overline{X}||^2$$

Where $\overline{X_k}$ is the centroid of cluster k, and, $\overline{X}$ is the mean of all samples.

We calculated the CI for the data projected onto the first 3 components of the cue-dependent subspace. First, we did this with the assumption of object-centered (position and size invariant) clustering (CI_{obj}, Supplementary Fig. 9a). Second, in every subspace that could be generated by the 10 PCs (from 1 to 10 dimensional subspaces, in total 1023), we calculated CI_{ego} based on the assumption of egocentric clustering. As a result, in all four cases of monkeys and brain areas, the CI_{obj} was smaller than all 1023 CI_{ego} samples (Supplementary Fig. 9b), which shows better decoding of the object-centered cue location than egocentric cue location.

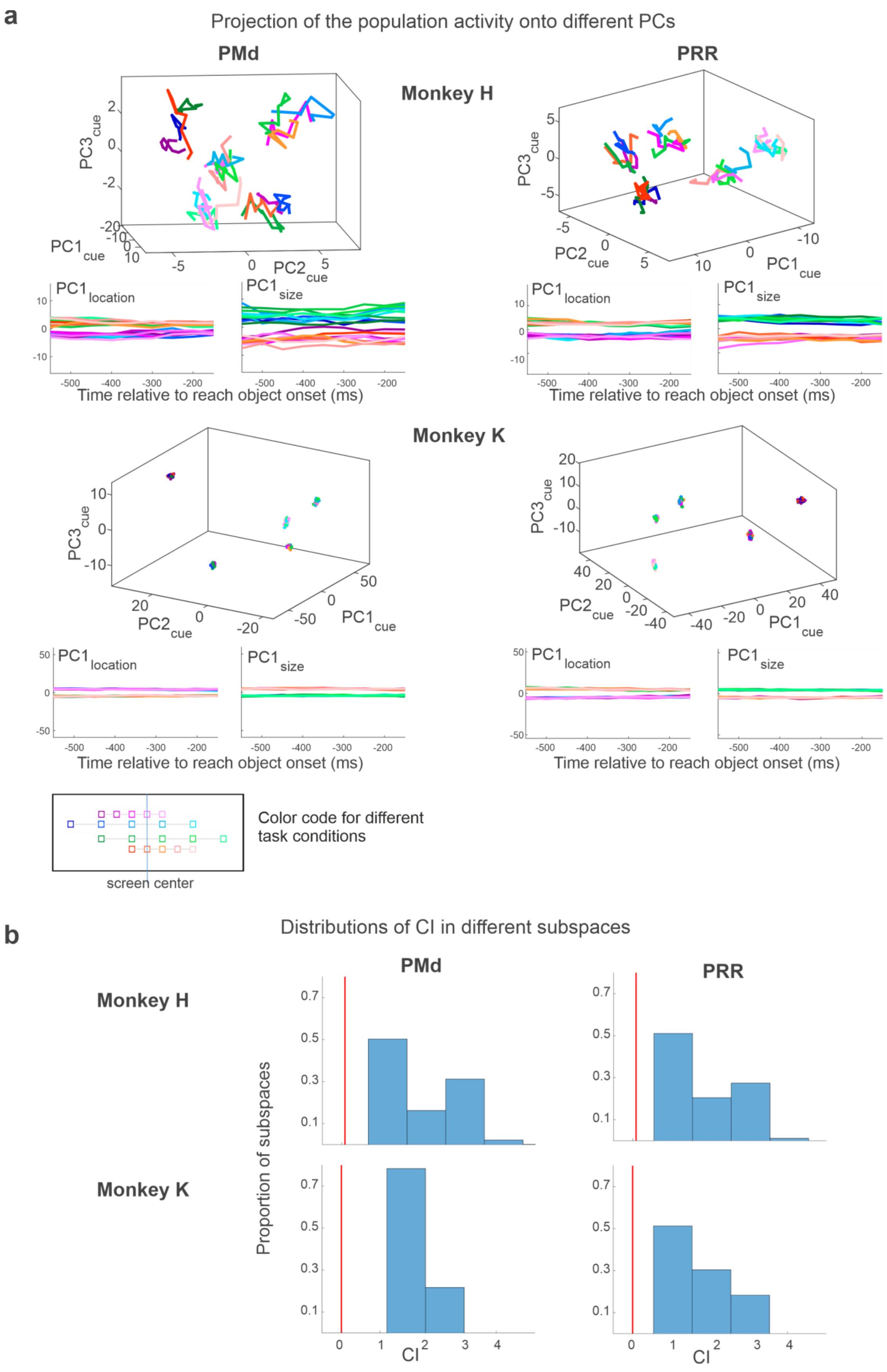
a
Projection of the population activity onto different PCs
PMd
PRR
Monkey H
Monkey K
PC1 location
PC1 size
PC1 cue
PC2 cue
PC3 cue
Time relative to reach object onset (ms)
Color code for different task conditions
screen center
b
Distributions of CI in different subspaces
PMd
PRR
Monkey H
Monkey K
Proportion of subspaces
CI

Supplementary Figure 9. Demixed Principal Component Analysis (dPCA) on Exp II data.

a Projection of the population activity from PMd (left column) and PRR (right column) onto the PCs that depend on different task parameters for monkeys H (top row) and K (bottom row). Each projection shows 20 neural trajectories corresponding to the 20 task conditions, color coded as follows (also graphically illustrated in the figure): object (location, size): (left, long) blue, (right, long) green, (left, short) purple, (right, short) orange. For each color, the five shades from dark to light represent the five boxes on the object from left to right. In each panel, the 3D plots show the data projected onto the first 3 components of the cue subspace. In both monkeys, 5 well-isolated clusters can be seen, each of which exclusively contains data that belongs to the same position of the cue on the object (as indicated by the color shading). In other words, the position of the cue on the object gives rise to the clustering and explains a large fraction of the variance in the data, while object size and position explain mostly the within-cluster variance. Below the 3D plot, left and right panels show the data projected onto the first object location PC and first object size PC, respectively. For the object size PC, task conditions with large object size (blue and green colors) make a cluster, and task conditions with short object size (purple and orange colors) make another cluster. A corresponding logic holds for the subspaces of object position. **b** Clustering index (CI). The distributions show the CI_{ego} calculated in 1023 subspaces of the first 10 PCs and the red line shows the CI_{obj} which was calculated in the first 3 dimensions of the cue-dependent subspace (the 3D plots in a). The CI_{obj} is always smaller than the CI_{ego} values, which indicates a better clustering of the object-centered compared to egocentric cue location.

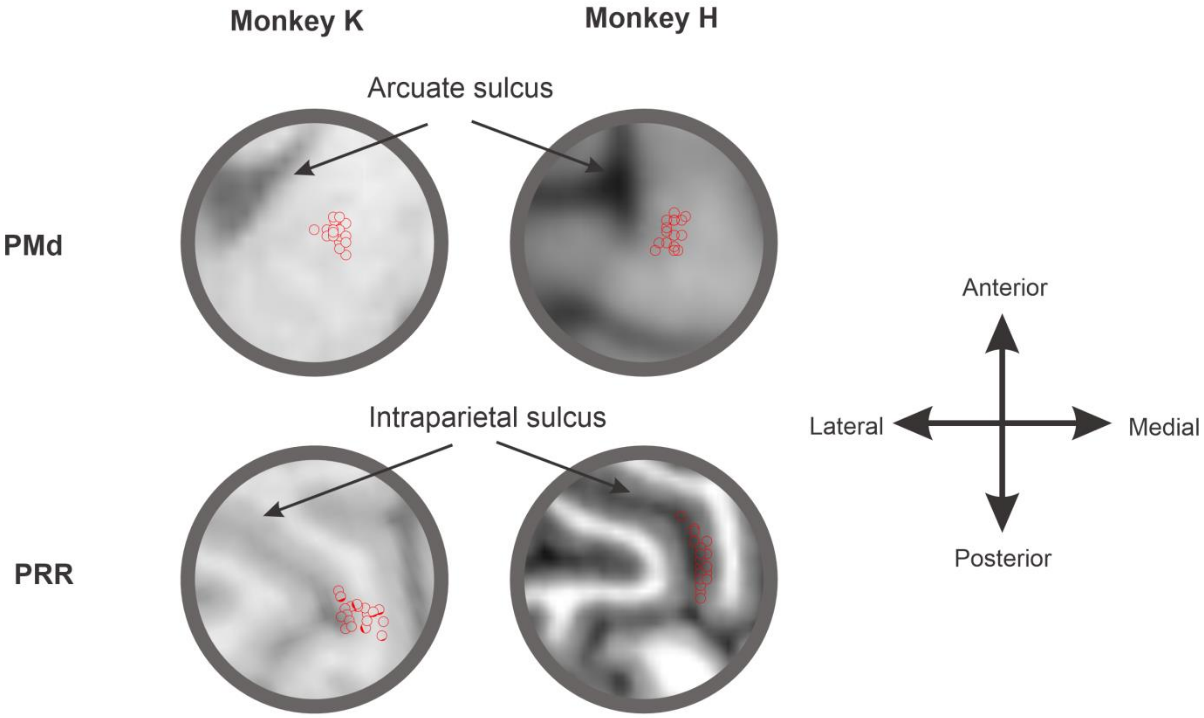

Supplementary Figure 10. Recording sites. Recording sites in PMd (top row) and PRR (bottom row) for monkey K (left column) and H (right column). Recording sites were reconstructed from a viewing angle perpendicular to the superficial cortical layer at the center of the chamber. PRR recording sites are presented in the depth of 6 mm for monkey K and 5 mm for monkey H, respectively, corresponding to the typical recording depth in these animals in PRR.

Supplementary Note 2: Animal implantation and neural recordings The procedures for animal preparation and neural recordings were described previously[2] and are here repeated for completeness. Numerical values have been adjusted to the current experiment. Two monkeys implanted with a titanium head holder and two magnetic resonance imaging (MRI)-compatible recording chambers, custom-fit to the monkeys' heads (3di, Jena Germany; and Thomas Recording, Giessen, Germany). Chamber positioning above PRR (Horsley Clarke coordinates: 10 mm contralateral and 13 mm posterior for monkey K; 12.5 mm contralateral and 13.5 mm posterior for monkey H) and PMd (18.5 mm contralateral and 20 mm anterior for monkey K; 19 mm contralateral and 22 mm anterior for monkey H) was guided by pre-surgical structural MRI and confirmed by postsurgical MRI. Sustained direction-selective neural responses during center-out reach planning (memory period) served as physiological signature to confirm the region of interest

in both areas. Supplementary Fig. 10 shows the recording sites on the MR images. Both chambers implanted contralaterally to the handedness of the monkey (left hemisphere). All surgical and imaging procedures were conducted under general anesthesia. We used two five-channel microdrives (“mini-matrix”; Thomas Recording) for extracellular recordings, mostly simultaneously in both chambers. The raw signals of the electrodes were pre-amplified (20x; Thomas Recording), band pass filtered, and amplified (154 Hz to 8.8 kHz; 400–800x; Plexon) before online spike sorting was conducted (Plexon Sort Client and server; Rasputin version 8.0). Spike times and spike waveforms were recorded and later subjected to additional offline sorting (Plexon Offline Sorter versions 3.3.5, 3.2.4 and 3.2.1).

Supplementary Table 1. Correlations and PI values for example neurons in Supplementary Fig. 3

Monkey K

	PMd, Late memory period		
	alloCorr	egoCorr	PI
Neuron 1	0.91	0.92	-0.02
Neuron 2	2.01	0.61	1.39
Neuron 3	1.68	1.23	0.45
Neuron 4	1.77	1.19	0.58
Neuron 5	1.33	0.54	0.79
Neuron 6	1.23	0.14	1.09
Neuron 7	2.37	0.98	1.39
Neuron 8	1.93	0.14	1.80
Neuron 9	1.28	1.15	0.13
Neuron 10	2.69	1.30	1.40
	PRR, Late memory period		
	alloCorr	egoCorr	PI
Neuron 1	1.87	1.75	0.12
Neuron 2	2.12	1.58	0.54
Neuron 3	1.35	-0.08	1.43
Neuron 4	2.56	0.10	2.45
Neuron 5	0.77	1.03	-0.26
Neuron 6	1.77	0.31	1.46
Neuron 7	1.48	-0.10	1.57
Neuron 8	1.49	0.60	0.89
Neuron 9	0.96	1.52	-0.56
Neuron 10	1.48	-0.22	1.70

	PMd, Late planning period		
	alloCorr	egoCorr	PI
Neuron 1	1.42	1.45	-0.03
Neuron 2	1.24	2.67	-1.43
Neuron 3	0.68	1.95	-1.27
Neuron 4	0.90	1.99	-1.09
Neuron 5	0.26	1.20	-0.94
Neuron 6	1.09	1.28	-0.18
Neuron 7	1.94	0.99	0.94
Neuron 8	0.28	2.76	-2.48
Neuron 9	1.63	3.02	-1.39
Neuron 10	1.13	3.86	-2.73
	PRR, Late planning period		
	alloCorr	egoCorr	PI
Neuron 1	1.11	3.18	-2.07
Neuron 2	2.93	0.78	2.15
Neuron 3	0.60	1.75	-1.14
Neuron 4	0.33	3.42	-3.09
Neuron 5	1.58	0.55	1.03
Neuron 6	1.18	0.83	0.35
Neuron 7	0.86	0.60	0.26
Neuron 8	-0.14	-0.04	-0.10
Neuron 9	1.31	2.34	-1.03
Neuron 10	0.68	3.05	-2.37

Monkey H

	PMd, Late memory period		
	alloCorr	egoCorr	PI
Neuron 1	1.12	-0.77	1.89
Neuron 2	1.72	1.00	0.71
Neuron 3	2.08	0.76	1.32
Neuron 4	1.86	1.90	-0.04
Neuron 5	1.88	1.22	0.65
Neuron 6	0.86	-0.19	1.05
Neuron 7	2.36	0.21	2.14
Neuron 8	1.06	3.54	-2.47
Neuron 9	0.84	-0.76	1.60
Neuron 10	2.09	2.22	-0.12
	PRR, Late memory period		
	alloCorr	egoCorr	PI
Neuron 1	1.14	0.54	0.60
Neuron 2	1.52	0.98	0.54
Neuron 3	1.67	2.02	-0.35

	PMd, Late planning period		
	alloCorr	egoCorr	PI
Neuron 1	1.14	0.54	0.60
Neuron 2	1.52	0.98	0.54
Neuron 3	1.67	2.02	-0.35
Neuron 4	-0.03	1.00	-1.03
Neuron 5	1.60	1.84	-0.24
Neuron 6	0.60	1.43	-0.83
Neuron 7	1.50	0.38	1.12
Neuron 8	0.89	0.15	0.74
Neuron 9	1.97	0.67	1.30
Neuron 10	2.24	1.28	0.97
	PRR, Late planning period		
	alloCorr	egoCorr	PI
Neuron 1	1.65	1.29	0.36
Neuron 2	1.53	0.43	1.10
Neuron 3	0.41	0.66	-0.25

Neuron 4	-0.03	1.00	-1.03
Neuron 5	1.60	1.84	-0.24
Neuron 6	0.60	1.43	-0.83
Neuron 7	1.50	0.38	1.12
Neuron 8	0.89	0.15	0.74
Neuron 9	1.97	0.67	1.30
Neuron 10	2.24	1.28	0.97

Neuron 4	3.32	1.24	2.07
Neuron 5	1.39	0.31	1.08
Neuron 6	0.96	0.77	0.19
Neuron 7	0.82	-0.72	1.53
Neuron 8	-0.55	1.84	-2.39
Neuron 9	1.20	0.76	0.44
Neuron 10	2.34	1.04	1.30

Supplementary References

1. Kobak, D. *et al.* Demixed principal component analysis of neural population data. *Elife.* **5**, (2016).

2. Westendorff, S., Klaes, C. & Gail, A. The cortical timeline for deciding on reach motor goals. *J.Neurosci.* **30**, 5426–5436 (2010).
