# Supplementary material for: Position- and scale-invariant object-centered spatial localization in monkey frontoparietal cortex dynamically adapts to cognitive demand
Source: Nat Commun. 2024 Apr 18;15:3357. doi: 10.1038/s41467-024-47554-4 (PMC11026390; doi:10.1038/s41467-024-47554-4)
Supplement: Supplementary file 3 — Reporting Summary [file 41467_2024_47554_MOESM3_ESM.pdf]

nature portfolio

Corresponding author(s): Alexander Gail

Last updated by author(s): Mar 6, 2024

Reporting Summary

Nature Portfolio wishes to improve the reproducibility of the work that we publish. This form provides structure for consistency and transparency in reporting. For further information on Nature Portfolio policies, see our Editorial Policies and the Editorial Policy Checklist.

Statistics

For all statistical analyses, confirm that the following items are present in the figure legend, table legend, main text, or Methods section.

n/a	Confirmed	
☐	☒	The exact sample size (n) for each experimental group/condition, given as a discrete number and unit of measurement
☐	☒	A statement on whether measurements were taken from distinct samples or whether the same sample was measured repeatedly
☐	☒	The statistical test(s) used AND whether they are one- or two-sided<br>*Only common tests should be described solely by name; describe more complex techniques in the Methods section.*
☐	☒	A description of all covariates tested
☐	☒	A description of any assumptions or corrections, such as tests of normality and adjustment for multiple comparisons
☐	☒	A full description of the statistical parameters including central tendency (e.g. means) or other basic estimates (e.g. regression coefficient) AND variation (e.g. standard deviation) or associated estimates of uncertainty (e.g. confidence intervals)
☐	☒	For null hypothesis testing, the test statistic (e.g. F, t, r) with confidence intervals, effect sizes, degrees of freedom and P value noted<br>*Give P values as exact values whenever suitable.*
☒	☐	For Bayesian analysis, information on the choice of priors and Markov chain Monte Carlo settings
☒	☐	For hierarchical and complex designs, identification of the appropriate level for tests and full reporting of outcomes
☐	☒	Estimates of effect sizes (e.g. Cohen's d, Pearson's r), indicating how they were calculated

Our web collection on statistics for biologists contains articles on many of the points above.

Software and code

Policy information about availability of computer code

Data collection	Plexon Sort Client and server (Rasputin version 2.6.0), and Offline Sorter (versions 3.3.5, 3.2.4 and 3.2.1) by Plexon were used for spike sorting. Experimental task and monitoring of monkey's behavior was controlled using a custom made software developed using LabVIEW (version 8.0).
Data analysis	Data was analyzed using custom made code developed in MATLAB 2016b and 2018b. Some of the results were plotted using the Gramm open source MATLAB toolbox. Code for reproducing figures and statistics can be found here: https://doi.org/10.25625/EO4DDY

For manuscripts utilizing custom algorithms or software that are central to the research but not yet described in published literature, software must be made available to editors and reviewers. We strongly encourage code deposition in a community repository (e.g. GitHub). See the Nature Portfolio guidelines for submitting code & software for further information.

Data

Policy information about availability of data

All manuscripts must include a data availability statement. This statement should provide the following information, where applicable:

- Accession codes, unique identifiers, or web links for publicly available datasets
- A description of any restrictions on data availability
- For clinical datasets or third party data, please ensure that the statement adheres to our policy

Data for reproducing figures and statistics can be found here: https://doi.org/10.25625/EO4DDY

Research involving human participants, their data, or biological material

Policy information about studies with human participants or human data. See also policy information about sex, gender (identity/presentation), and sexual orientation and race, ethnicity and racism.

Reporting on sex and gender	N/A
Reporting on race, ethnicity, or other socially relevant groupings	N/A
Population characteristics	N/A
Recruitment	N/A
Ethics oversight	N/A

Note that full information on the approval of the study protocol must also be provided in the manuscript.

Field-specific reporting

Please select the one below that is the best fit for your research. If you are not sure, read the appropriate sections before making your selection.

☒ Life sciences ☐ Behavioural & social sciences ☐ Ecological, evolutionary & environmental sciences

For a reference copy of the document with all sections, see nature.com/documents/nr-reporting-summary-flat.pdf

Life sciences study design

All studies must disclose on these points even when the disclosure is negative.

Sample size	Two animal subjects were used for this study which is standard in the field. No statistics are conducted on this N=2, but on the number of recorded neurons/single units. In total, we recorded 100 and 107 single units from PRR and PMd in experiment I, 103 and 98 single units from PRR and PMd in experiment II, all sample sizes are within the range of average sample sizes reported in previous studies. (examples of previous studies with the sample size within this range: https://doi.org/10.7554/eLife.51322; https://doi.org/10.7554/eLife.47581; https://doi.org/10.1016/j.neuron.2011.02.053)
Data exclusions	Neurons were selected based on pre-defined, standard, electrophysiological activity within the trial, as detailed in the manuscript. This will ensure that only those neurons which were actively engaged in the experiment were included in the analysis, not those which were only active between trials and became silent as the behavioral task began.
Replication	Results were replicated in two animals (rhesus monkeys), as is typical and accepted standard in this field of research.
Randomization	Experimental conditions were randomized in every session to eliminate any bias due to predictive activities in the neural data. Multiple single units were sampled in every session to collect a random sample from each brain area across sessions.
Blinding	Blinding is not relevant because all the results were driven from computerized, non-biased statistical analyses. The exclusion criteria for neurons are independent from the phenomena being investigated, which is standard in the field.

Reporting for specific materials, systems and methods

We require information from authors about some types of materials, experimental systems and methods used in many studies. Here, indicate whether each material, system or method listed is relevant to your study. If you are not sure if a list item applies to your research, read the appropriate section before selecting a response.

Materials & experimental systems

n/a	Involved in the study	
☒	☐	Antibodies
☒	☐	Eukaryotic cell lines
☒	☐	Palaeontology and archaeology
☐	☒	Animals and other organisms
☒	☐	Clinical data
☒	☐	Dual use research of concern
☒	☐	Plants

Methods

n/a	Involved in the study	
☒	☐	ChIP-seq
☒	☐	Flow cytometry
☒	☐	MRI-based neuroimaging

Animals and other research organisms

Policy information about studies involving animals; ARRIVE guidelines recommended for reporting animal research, and Sex and Gender in Research

Laboratory animals	Two adult male rhesus monkeys (Macaca mulatta) of age 11 and 9 years old.
Wild animals	Wild animals were not used in this study.
Reporting on sex	Two adult male rhesus monkeys (Macaca mulatta)
Field-collected samples	No field-collected samples were used in the study.
Ethics oversight	All procedures have been approved by the responsible regional government office [Niedersächsisches Landesamt für Verbraucherschutz und Lebensmittelsicherheit (LAVES)] under permit numbers 3392 42502-04-13/1100 and comply with German Law and the European Directive 2010/63/EU regulating use of animals in research.

Note that full information on the approval of the study protocol must also be provided in the manuscript.

Plants

Seed stocks	*Report on the source of all seed stocks or other plant material used. If applicable, state the seed stock centre and catalogue number. If plant specimens were collected from the field, describe the collection location, date and sampling procedures.*
Novel plant genotypes	*Describe the methods by which all novel plant genotypes were produced. This includes those generated by transgenic approaches, gene editing, chemical/radiation-based mutagenesis and hybridization. For transgenic lines, describe the transformation method, the number of independent lines analyzed and the generation upon which experiments were performed. For gene-edited lines, describe the editor used, the endogenous sequence targeted for editing, the targeting guide RNA sequence (if applicable) and how the editor was applied.*
Authentication	*Describe any authentication procedures for each seed stock used or novel genotype generated. Describe any experiments used to assess the effect of a mutation and, where applicable, how potential secondary effects (e.g. second site T-DNA insertions, mosiacism, off-target gene editing) were examined.*
